# Effects of acute strawberry consumption on serum levels of vitamin C and folic acid, the antioxidant potential of LDL and blood glucose response: a randomised cross-over controlled trial

**DOI:** 10.1017/jns.2022.117

**Published:** 2023-03-22

**Authors:** Yoshimi Kishimoto, Chie Taguchi, Tomomi Iwashima, Toshihiro Kobayashi, Yutaka Kikoku, Hiroshi Nishiyama, Yasunobu Masuda, Kazuo Kondo

**Affiliations:** 1 Department of Food Science and Human Nutrition, Faculty of Agriculture, Setsunan University, 45-1 Nagaotouge-cho, Hirakata, Osaka 573-0101, Japan; 2 National Institute of Health Sciences, 3-25-26 Tonomachi, Kawasaki-ku, Kawasaki, Kanagawa 210-9501, Japan; 3 Ochanomizu University, 2-1-1 Otsuka, Bunkyo-ku, Tokyo 112-8610, Japan; 4 R&D Division, AOHATA Corporation, 1-1-25 Tadanouminakamachi, Takehara-shi, Hiroshima 729-2392, Japan; 5 R&D Division, Kewpie Corporation, 2-5-7 Sengawa-cho, Chofu-shi, Tokyo 182-0002, Japan

**Keywords:** Blood glucose level, Folate, LDL oxidation, Strawberry, Vitamin C

## Abstract

Strawberry contains many bioactive compounds such as vitamin C and polyphenols as well as folate, a vitamin that is especially important for women of childbearing age. We investigated the effects of the acute consumption of strawberry on the serum levels of vitamin C and folate, and on the antioxidant potential of low-density lipoprotein (LDL). In a randomised, placebo-controlled, double-blind, crossover study, twenty-three healthy female volunteers (age 22⋅5 ± 1⋅4 years) ingested 500 g of a strawberry purée beverage or a sugar content-matched placebo beverage. Blood samples were collected at fasting and at 0⋅5, 1, 2 and 4 h post-ingestion. The serum concentrations of vitamin C and folate were significantly elevated from 0⋅5 to 4 h after the strawberry beverage ingestion (*P* < 0⋅001); the levels peaked at 2 h, with peak levels of 15⋅0 ± 2⋅5 μg/ml for vitamin C and 14⋅4 ± 7⋅0 ng/ml for folate. Notably, at 1 h after the strawberry beverage ingestion, the LDL oxidation lag time was significantly prolonged (*P* < 0⋅05), suggesting that the antioxidant potential of LDL was increased. After the ingestion of either beverage, the serum levels of glucose and insulin reached a peak at 0⋅5 h and then quickly returned to baseline levels. These results suggest that strawberries are a useful source of vitamin C and folate and may help enhance the antioxidant potential of LDL in healthy young women.

## Introduction

Many epidemiological studies have shown that a high consumption of fruits and vegetables is associated with a reduced risk of cardiovascular disease (CVD) and other chronic diseases^(1–3)^. Fruits and vegetables contain many components that contribute to human health, including vitamins, fibre, carotenoids, sulphur compounds, nitrate and organic acids, as well as a wide variety of polyphenolic compounds^(4)^. These are likely to act synergistically through multiple biological mechanisms to reduce the risk of the development of chronic diseases. Japan's national health promotion policy guidelines, i.e. ‘Health Japan 21’ (the second version), which were established to improve the lifestyle and extend the healthy life expectancy of Japan's population, set a target of reducing the percentage of individuals who consume <100 g of fruit per day to <30 % by 2022^(5)^. However, the intake of fruit by Japanese individuals is still at a low level, with the average per capita fruit intake reported to be 96⋅4 g/d in 2019^(6)^. A recent scenario analysis indicated that there is a significant potential for reducing the burden of CVD in Japan by increasing the population's fruit intake^(7)^.

Strawberries are consumed worldwide and are important sources of nutritive compounds such as vitamin C, folate and polyphenols. Strawberries are one of the most abundant sources of folate among fruits^(8)^. Folates play a key role in one-carbon metabolism, which involves nucleotide biosynthesis, DNA and amino acid synthesis, and the re-methylation of homocysteine to methionine^(9)^. An increased plasma homocysteine concentration is a risk factor for CVD^(10,11)^. Moreover, a deficiency of folate post-conception and during early pregnancy can result in neural tube defects in newborns^(12)^. The polyphenols that accumulate in ripe strawberries include flavonoids, which include anthocyanins, flavonols and flavan-3-ols, as well as ellagitannins and phenolic acids^(13,14)^, all of which are antioxidants.

Clinical intervention studies revealed that a regular consumption of strawberries may positively affect risk factors for CVD. Total cholesterol (TC) and low-density lipoprotein (LDL) cholesterol levels were shown to be decreased by strawberry supplementation in overall healthy adults with abdominal obesity and dyslipidemia^(15,16)^. The consumption of strawberries improved healthy subjects’ plasma lipids profile, biomarkers of antioxidant status, antihemolytic defenses and platelet function^(17)^. Strawberry supplementation was also associated with increased serum antioxidant capacity in healthy volunteers^(18,19)^. Notably, the postprandial responses of serum triglyceride and oxidised LDL levels among overweight hyperlipidemic subjects after a high-fat meal were attenuated by strawberry consumption^(20)^. It is well established that an increased susceptibility of LDL to oxidative modification promotes the formation of atherosclerosis. LDL particles are protected against oxidants by antioxidants (e.g. α-tocopherol) that are present in LDL particles and thus resistant to oxidative modification^(21)^. We hypothesised that after strawberries are consumed, antioxidants in the strawberries might interact with LDL and protect LDL from reactive oxygen species.

However, the above-cited studies were all long-term intervention trials and did not examine the acute impacts of strawberry consumption on vitamins’ postprandial response or on antioxidant capacity. Another issue to consider is the relatively high sugar content of fruits marketed in Japan, which often raises concerns about the fruits’ effect on the blood glucose level. We thus conducted the present study to determine how acute strawberry consumption affects serum levels of vitamin C and folic acid, the antioxidant potential of LDL and the blood glucose response.

## Subjects and methods

### Subjects

Healthy female volunteers aged 20–35 years were recruited by a public recruitment poster, with the following exclusion criteria: (1) allergy to strawberries, (2) smoking habit, (3) taking supplements such as vitamins, (4) having disorderly dietary habits, (5) current participation in another clinical study and (6) evaluated as ineligible by a physician. The study was conducted according to the guidelines laid down in the Declaration of Helsinki and all procedures involving human subjects were approved by the Ethics Committee of Ochanomizu University (2017–16). Written informed consent for participation was obtained from all subjects. The study was registered in the UMIN-CTR as UMIN000031855 (https://www.umin.ac.jp/ctr/).

### Experimental design and diets

We conducted a randomised, placebo-controlled, double-blind, crossover trial. Based on a previous study^(22)^, a minimum sample size of nineteen participants was required to detect a difference in the LDL lag time with >80 % power with a probability (*P*)-value < 0⋅05. Considering a drop-out rate of 20 %, twenty-three subjects were recruited. The subjects were randomly allocated into one of two groups using computer-generated random numbers by a researcher unrelated to this study. The participants were asked to complete two sessions in which a strawberry purée beverage (500 g) or a sugar content-matched placebo beverage (500 g) was ingested, in a randomised order. The test days were separated by a 4-week washout period. The subjects’ consumption of strawberries was restricted during the intervention period. Strawberry fruits of the cultivar ‘*Yumetsuzuki*’, created by Japan's National Agriculture and Food Research Organization and AOHATA Corp. (Takehara, Japan), were selected for the study because of their high anthocyanins content. The nutritional composition of the strawberry and placebo beverages per serving are summarised in Table 1. The strawberry purée beverage contained 500 g/serving, which delivered 16 g glucose, 16 g fructose, 442⋅5 mg total polyphenolic compounds, mainly anthocyanins (171⋅5 mg), with pelargonidin-3-glucoside being the most dominant component (136⋅5 mg), 255 mg vitamin C (total ascorbic acid) and 330 μg folate. The placebo beverage was sugar content-matched and flavoured to feel identical to the strawberry beverage, without the phytochemical composition. Both beverages were formulated in a single batch by AOHATA Corp. and were provided in dark packages. The beverages were stored at −20°C before use.

**Table 1.** Nutritional composition of the strawberry and placebo beverages per serving

				Strawberry beverage	Placebo beverage
				(per 500 g)	(per 500 g)
Energy			(kcal)	195	128
Protein			(g)	3⋅5	–
Fat			(g)	0⋅5	–
Carbohydrate			(g)	44⋅5	–
	Available		(g)	38⋅5	–
		Sucrose	(g)	N.D.	–
		Glucose	(g)	16	16
		Fructose	(g)	16	16
	Fibre		(g)	6	–
Polyphenols	Total		(mg)	442⋅5	–
	Anthocyanins		(mg)	171⋅5	–
		Cyanidin-3-glucoside	(mg)	4	–
		Pelargonidin-3-glucoside	(mg)	136⋅5	–
		Pelargonidin-3-galactoside	(mg)	17	–
		Pelargonidin	(mg)	14	–
	Catechin		(mg)	9⋅5	–
	Ellagic acid		(mg)	60	–
Vitamin C (total ascorbic acid)			(mg)	255	–
Folate			(μg)	330	–

### Blood sampling and measurements

The subjects attended the two sessions at the laboratory in the morning between 8:00 and 9:00 am, 4 weeks apart. They were asked to consume a standardised meal box by 8:00 pm the night before the test and to not eat or drink anything other than water after that. Blood samples were collected from the subjects before and at 0⋅5, 1, 2 and 4 h after consuming either beverage. Blood glucose concentrations were measured by the hexokinase UV method. Serum insulin and folate concentrations were measured by a chemiluminescent enzyme immunoassay. Serum vitamin C (total ascorbic acid) was measured by high-performance liquid chromatography. TC, high-density lipoprotein cholesterol (HDL-C), triglyceride (TG) and non-esterified fatty acid (NEFA) were measured by enzymatic assays. LDL cholesterol (LDL-C) was calculated using the Friedewald formula. The above biochemistry tests were performed by SRL Inc. (Tokyo).

### LDL oxidation lag time assay

LDL was separated from each subjects’ plasma by single-spin density gradient ultracentrifugation at 100 000 rpm for 40 min at 4°C. The LDL oxidizability was determined by a lag time assay as described^(22)^. The prepared LDL samples (final concentration of protein: 70 μg/ml) were oxidised by 200 μM 2,2-azobis-4-methoxy-2,4-dimethylvaleronitrile (Wako Pure Chemicals, Osaka, Japan). The kinetics of LDL oxidation were determined by monitoring the absorbance of conjugated dienes at 234 nm with a spectrophotometer (DU800, Beckman Coulter, Indianapolis, IN, USA) at 4-min intervals at 37°C. The lag time for starting LDL oxidation was defined as the time interval between the initiation and the intercept of the two tangents drawn to the lag and the propagation phase of the absorbance curve at 234 nm.

### Statistical analyses

All data are expressed as the mean ± standard deviation (sd). A two-way repeated measures analysis of variance (ANOVA) and *post-hoc* analysis were performed to test the postprandial changes in the variables. When the interaction was significant, a simple main-effects test with Bonferroni adjustment was selected. Values of *P* < 0⋅05 were considered significant. All statistical analyses were conducted with IBM SPSS software, ver. 26.

## Results

**Fig. 1.**
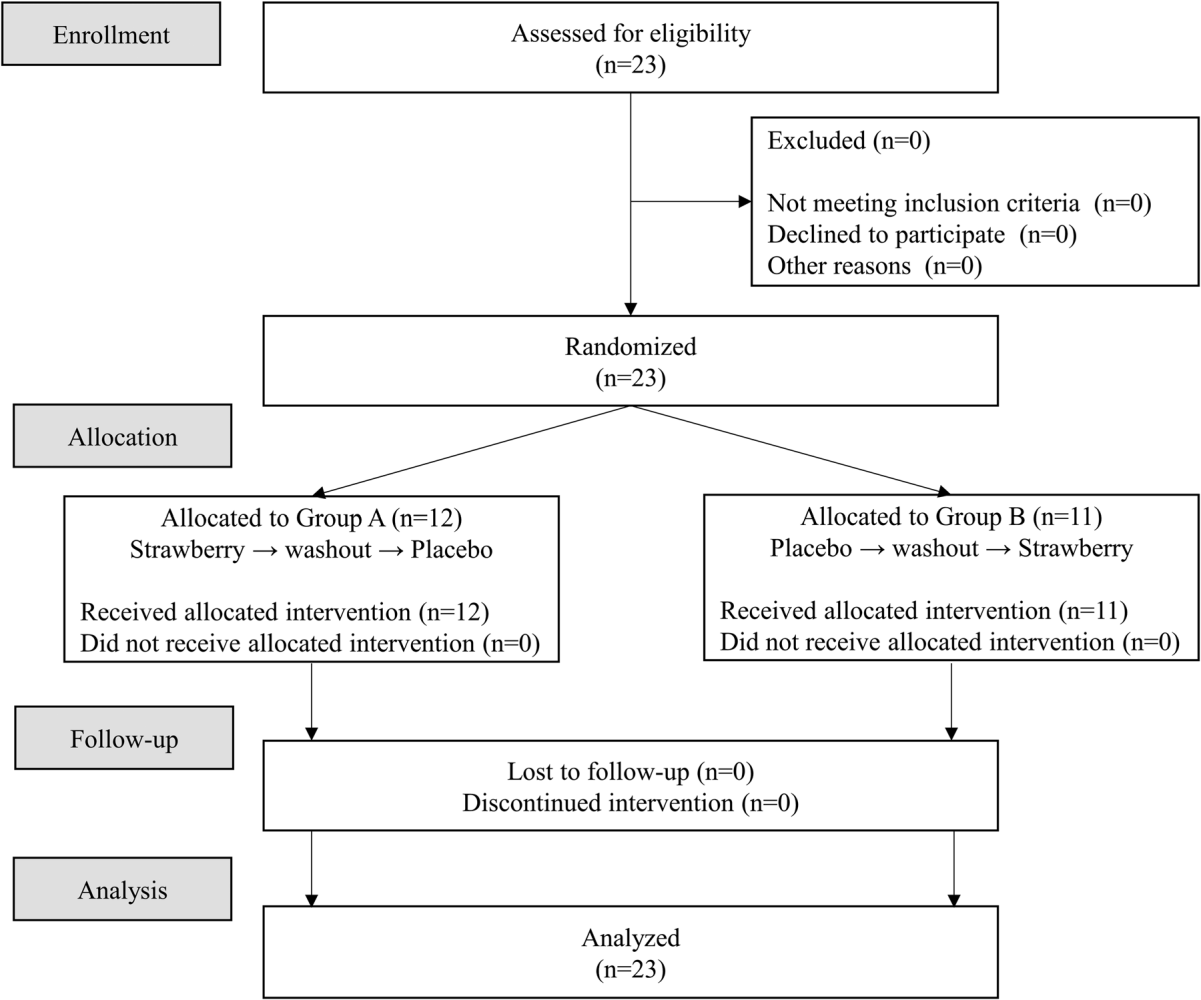
A CONSORT diagram outlining the flowchart of the study. The study was conducted with twenty-three healthy women in a randomised, placebo-controlled, double-blind, crossover design.

A CONSORT diagram outlining the flowchart of the study is provided in Fig. 1. Twenty-three subjects were recruited, and all subjects who underwent randomisation completed the two sessions. Data from all twenty-three subjects were included the analyses. The characteristics of the subjects are summarised in Table 2. The mean age was 22⋅5 ± 1⋅4 years and the body mass index (BMI) was 21⋅3 ± 2⋅2 kg/m^2^. No effect of the order of interventions was observed, suggesting that the test was considered to be appropriate based on crossover methods.

**Table 2.** Baseline characteristics of the subjects (*n* 23)

		Mean	(sd)
Age	(years)	22⋅5	(1⋅4)
Height	(cm)	157⋅1	(4⋅6)
Weight	(kg)	52⋅7	(7⋅4)
BMI	(kg/m^2^)	21⋅3	(2⋅2)
SBP	(mmHg)	112	(7)
DBP	(mmHg)	65	(7)

BMI, body mass index; SBP, systolic blood pressure; DBP, diastolic blood pressure.

**Fig. 2.**
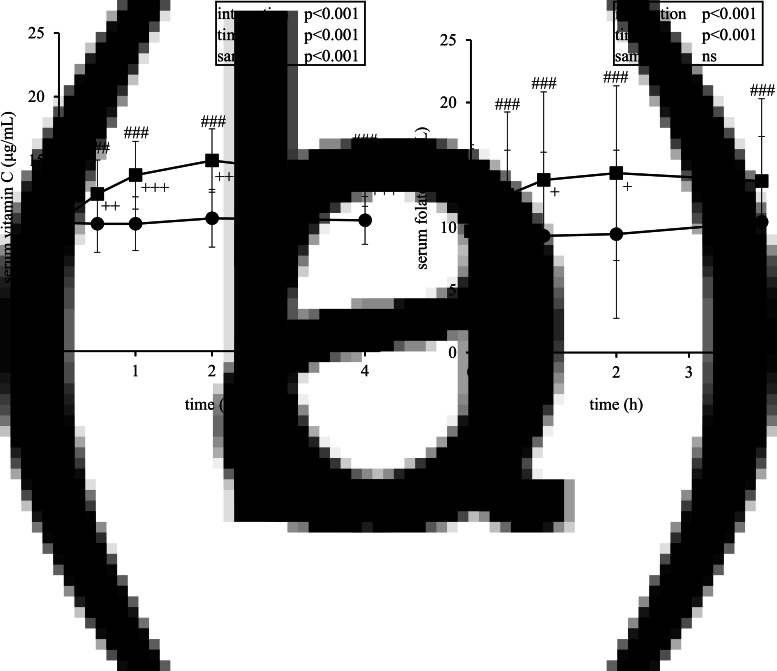
Postprandial changes in serum vitamin C (a) and folate level (b) after the ingestion of the placebo (*circles*) or strawberry (*squares*) beverage. The data are mean ± sd (*n* 23). ^+^*P* < 0⋅05, ^++^*P* < 0⋅01, ^+++^*P* < 0⋅001 *v*. placebo, ^###^*P* < 0⋅001 *v*. 0 h by pairwise comparisons with Bonferroni corrections.

After the ingestion of the strawberry beverage, the serum concentrations of vitamin C and folate were significantly increased from 0⋅5 to 4 h (*P* < 0⋅001, Fig. 2). The levels peaked at 2 h, with peak levels of 15⋅0 ± 2⋅5 μg/ml for vitamin C and 14⋅4 ± 7⋅0 ng/ml for folate.

**Fig. 3.**
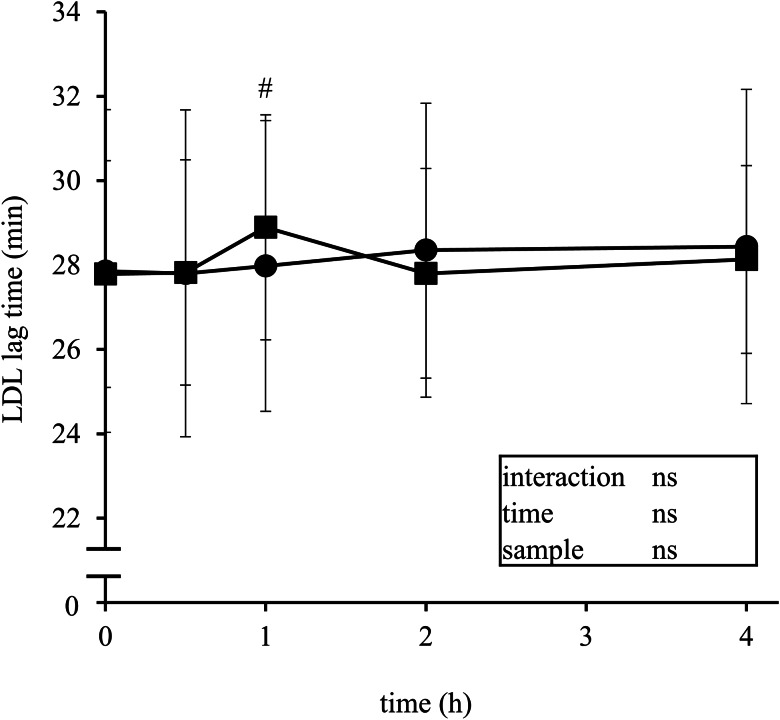
Postprandial changes in the LDL lag time after the ingestion of placebo (*circles*) or strawberry (*squares*) beverage. The data are mean ± sd (*n* 21). ^#^*P* < 0⋅05 *v*. 0 h by Dunnett's multiple comparison test after 1-way ANOVA.

The LDL oxidation lag time for the initiation of conjugated diene formation was significantly prolonged at 1 h after the ingestion of the strawberry beverage compared to that before the strawberry intake (29⋅0 ± 3⋅6 min *v*. 28⋅0 ± 3⋅6 min, *P* < 0⋅05, Fig. 3). In contrast, no significant change in the LDL lag time was observed after the ingestion of the placebo beverage.

**Fig. 4.**
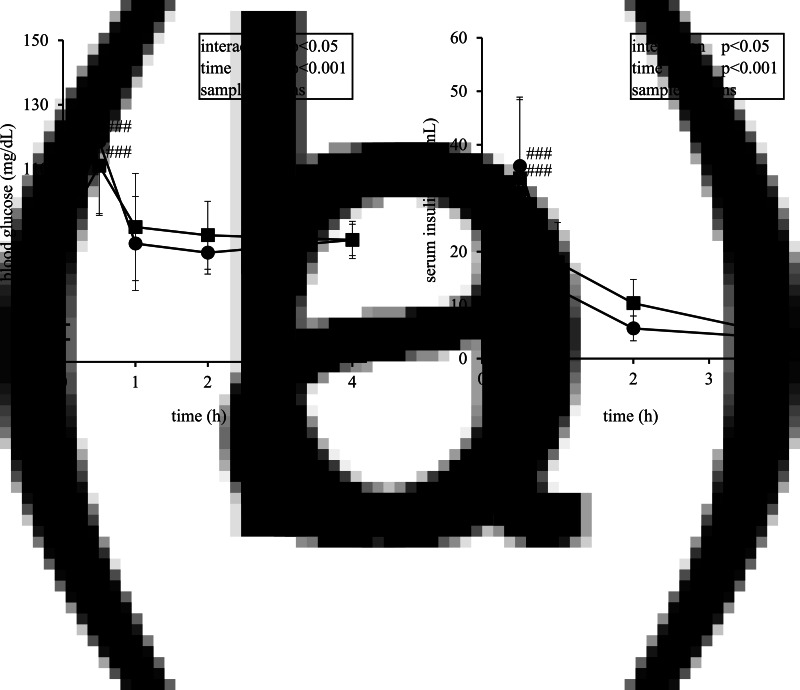
Postprandial changes in blood glucose (a) and the serum insulin level (b) after the ingestion of placebo (*circles*) or strawberry (*squares*) beverage. The data are mean ± sd (*n* 23). ^###^*P* < 0⋅001 *v*. 0 h by pairwise comparisons with Bonferroni corrections.

The serum concentrations of glucose reached a peak at 0⋅5 h after the ingestion of either beverage. The changes in peak glucose levels were slightly lower when the strawberry beverage was ingested compared with the placebo, but the difference did not reach significance (20⋅3 ± 13⋅8 mg/dl *v*. 27⋅6 ± 20⋅5 mg/dl, Fig. 4(a)). The insulin response after ingestion behaved similarly to that of blood glucose, reaching a peak level at 0⋅5 h and then returning to baseline (Fig. 4(b)).

The acute ingestion of the strawberry beverage (500 g) did not cause an elevation of serum lipids (Table 3). With both beverages, there were no notable differences in the variability of TC, HDL-C, LDL-C, TG and NEFA in the postprandial state.

**Table 3.** Postprandial changes in serum lipids after the ingestion of the placebo or strawberry beverage

	Placebo										Strawberry									
	0 h		1 h		2 h		3 h		4 h		0 h		1 h		2 h		3 h		4 h	
	Mean	(sd)	Mean	(sd)	Mean	(sd)	Mean	(sd)	Mean	(sd)	Mean	(sd)	Mean	(sd)	Mean	(sd)	Mean	(sd)	Mean	(sd)
TC (mg/dl)	189⋅3	(35⋅1)	178⋅2	(29⋅7)*	183⋅3	(30⋅5)*	181⋅9	(29⋅9)*	182⋅1	(30⋅7)*	189⋅1	(35⋅8)	182	(32⋅6)*	183⋅6	(34⋅3)*	186⋅4	(34⋅7)	186	(32⋅3)
HDL-C (mg/dl)	72⋅9	(15⋅9)	69⋅9	(14⋅9)*	71⋅6	(14⋅6)	70⋅6	(15⋅3)*	70⋅2	(15⋅6)*	75⋅4	(19⋅4)	73⋅4	(19⋅1)*	74⋅1	(20)	75⋅1	(20)	74⋅1	(19)
LDL-C (mg/dl)	101	(22)	94⋅1	(19⋅0) *	97⋅6	(21⋅1) *	98⋅2	(20⋅2) *	97⋅2	(20⋅4) *	100⋅5	(22⋅5)	96⋅3	(20⋅3) *	96⋅9	(21⋅1) *	99⋅5	(21⋅5)	98⋅7	(21⋅2)
TG (mg/dl)	76⋅7	(57⋅1)	71⋅3	(47⋅5)	70⋅8	(47⋅5)	65⋅7	(43⋅1)*	73⋅6	(42⋅1)	65⋅9	(30⋅9)	61⋅7	(29⋅8)	62⋅6	(27⋅5)	59	(27⋅0)*	66	(25⋅1)
NEFA (μU/ml)	539⋅3	(265⋅9)	178⋅1	(122⋅2)*	87⋅7	(31⋅7)*	268⋅7	(190⋅5)*	546⋅3	(200⋅8)	518⋅3	(184⋅4)	211⋅6	(90⋅5)*	91⋅3	(37⋅6)*	126	(75⋅9)*	478⋅5	(181⋅4)

* *P* < 0⋅05 *v*. 0 h by Dunnett's multiple comparison test after 1-way ANOVA.

## Discussion

The present results demonstrated that an acute intake of a strawberry beverage increased serum vitamin C and folate levels and enhanced the antioxidant capacity of LDL in healthy young women. There was no significant difference in the glycemic response between the placebo and strawberry beverage consumptions. This is one of the few studies that have investigated the effect of an acute intake of a food product that is rich in folate and/or vitamin C on postprandial changes in their serum levels.

Several clinical studies have indicated a great variation in the bioavailability of food folate (ranging from 30 to 98 % compared with chemical-based folic acid products)^(23,24)^. The bioavailability of food folates is dependent on the food matrix, the degree of folate release from the matrix and the presence of other food constituents such as ascorbic acid^(25)^. Striegel *et al.* demonstrated that folate from strawberries is highly bioavailable and absorbable by the human body^(26)^. In the present study, after the subjects’ consumption of the strawberry beverage, the serum concentrations of total folate increased rapidly from 0⋅5 h and remained high until 4 h later. The World Health Organization (WHO) proposed that serum total folate values ≤4⋅0 ng/ml indicate a folate deficiency^(27)^. Ihara *et al.* reported that in fifty-eight healthy Japanese women, the 95 % distribution range of serum total folate concentrations was 5⋅2–19⋅5 ng/ml, and the median value was 9⋅7 ng/ml^(28)^. In our present study, the median value of serum total folate at baseline was 7⋅7 ng/ml (min.–max.: 3⋅5–31⋅4 ng/ml). Although the effects of a long-term intake of strawberries have not been studied, strawberry consumption may be useful as a source of dietary folate. Strålsjö *et al.* reported that the total folate content in thirteen different Swedish strawberry cultivars varied from 30 to 69 μg/100 g of fresh weight^(29)^. The analysed value of the folate content in the cultivar *Yumetsuzuki* is 66 μg/100 g, which is in the top rank compared with the report by Strålsjö *et al.* The folate content of strawberry (raw) listed in the Standard Tables of Food Composition in Japan is 90 μg/100 g^(30)^, indicating that Japanese strawberry cultivars are generally rich in folate.

Our present analyses also revealed that the ingestion of the strawberry beverage significantly extended the lag phase of LDL oxidation. This acute effect against LDL oxidation might be due to the antioxidant properties of the phytonutrients of strawberry. Another study showed that the LDL oxidation in response to a high-fat meal was attenuated after 6 weeks of the daily consumption of a strawberry beverage^(20)^. It was also reported that a 1-month strawberry supplementation reduced oxidative damage to LDL measured as thiobarbituric acid-reactive substances in the LDL fraction^(31)^. A possible explanation for that findings is that phenolic compounds bind to LDL and protect LDL from reactive oxygen species through their oxidants scavenging activity.

Our earlier study demonstrated that an acute intake of green tea catechins at a dose of 1 g total catechin (of which most [>99 %] was the gallated type) resulted in increases in the levels of LDL catechins and prolonged the lag time of LDL oxidation in healthy adults^(22)^. Our group also showed that the consumption of grapes containing 1 g total polyphenolic compounds prolonged the lag time of LDL oxidation^(32)^. In that study, the antioxidant activity of grape components and metabolites was examined by a lag time assay and a radical scavenging assay *in vitro*. Cyanidin-3-glucoside, protocatechuic acid, resveratrol and ascorbic acid prolonged the lag time, indicating that these components may protect LDL from oxidative modification. The strawberry beverage used in the present investigation contained 442⋅5 mg of total polyphenols (mainly anthocyanins, with pelargonidin-3-glucoside as the major component) and 225 mg of ascorbic acid. Although the amounts of polyphenolic compounds and ascorbic acid in the present test beverage were lower than those described as effective in the previous reports, these components might cooperatively inhibit LDL oxidation. Safari *et al.* reported that an incubation of plasma with pelargonidin caused it to be incorporated into LDL particles, making them less susceptible to oxidative modification *in vitro*^(33)^. To clarify whether pelargonidin can bind to the LDL fraction after strawberry consumption, a future study should measure the concentration of pelargonidin in the LDL fraction.

We also observed that the subjects’ serum concentrations of glucose reached a peak at 0⋅5 h after the ingestion of either the strawberry or placebo beverage. There was no significant difference in peak blood glucose levels between the placebo and strawberry ingestions. The consumption of 16 g of glucose and 16 g of fructose from the strawberry beverage caused slight increases in blood glucose and insulin at 0⋅5 h, and the values then quickly returned to baseline levels, which was similar to the placebo beverage. It has been reported that dietary supplementation with strawberries improved postprandial insulin responses, blunting postprandial insulin and glucose responses in both metabolically healthy and unhealthy subjects (e.g. subjects with obesity, metabolic syndrome or type 2 diabetes mellitus)^(34)^. Anthocyanins have also been shown to suppress glucose transport from the intestine to plasma, specifically by inhibiting the sodium glucose co-transporter 1 (SGLT1) and the glucose transporter GLUT2^(35)^. In the present investigation, only a small amount of the sugars in strawberries was consumed, and thus the increase in the blood glucose level was mild and not significantly different from that produced by the placebo.

Our study has several limitations. First, individual genetic diversity such as single nucleotide polymorphisms of methylenetetrahydrofolate reductase were not taken into account. Second, we measured postprandial changes in serum vitamin C and folate levels, but not changes in polyphenolic compounds. Third, the antioxidant capacity of LDL was evaluated only by the *ex vivo* lag time assay, and due to the limited sample volume, multiple measurements could not be performed. Last, the test dates were spaced 4 weeks apart to account for the subjects’ menstrual cycles, but hormones may have had some effect on the results. In addition, the study was of healthy women aged 20–35 years, who are generally considered to be at low risk for CVD, and these results may not be applicable to other populations.

In conclusion, a single intake of a strawberry beverage increased serum vitamin C and folate levels and enhanced the antioxidant capacity of LDL, and this can be expected to contribute to the health of women of childbearing age.
